# Study of Two Bovine Bone Blocks (Sintered and Non-Sintered) Used for Bone Grafts: Physico-Chemical Characterization and In Vitro Bioactivity and Cellular Analysis

**DOI:** 10.3390/ma12030452

**Published:** 2019-02-01

**Authors:** Sergio Alexandre Gehrke, Patricia Mazón, Leticia Pérez-Díaz, José Luis Calvo-Guirado, Pablo Velásquez, Juan Manuel Aragoneses, Manuel Fernández-Domínguez, Piedad N. De Aza

**Affiliations:** 1 Department of Research, Biotecnos, Cuareim 1483, CP 11100 Montevideo, Uruguay; sergio.gehrke@hotmail.com; 2 Department of Materiales, Óptica y Tecnología Electrónica, Universidad Miguel Hernández, Avda, Universidad s/n, 03202 Elche (Alicante), Spain; pmazon@umh.es; 3 Laboratorio de Interacciones Molecular, Facultad de Ciencias, Universidad de la Republica, Calle Iguá 4225, 11400 Montevideo, Uruguay; letperez@gmail.com; 4 Department of Oral and Implant Surgery, Faculty of Health Sciences, Universidad Católica de Murcia (UCAM), 30107 Murcia, Spain; jlcalvo@ucam.edu; 5 Instituto de Bioingenieria, Universidad Miguel Hernández, Avda, Ferrocarril s/n, 03202 Elche (Alicante), Spain; pavelasquez@umh.es; 6 Department of Dental Research in Universidad Federico Henriquez y Carvajal (UFHEC), Santo Domingo 10107, Dominican Republic; jaragoneses@ufhec.edu.do; 7 Department of Translational Medicine, CEU San Pablo University, 28223 Madrid, Spain; clinferfun@yahoo.es

**Keywords:** ceramic blocks, bone graft, bovine bone, biomaterials, cellular analysis

## Abstract

In this work, the physicochemical properties and in vitro bioactivity and cellular viability of two commercially available bovine bone blocks (allografts materials) with different fabrication processes (sintered and not) used for bone reconstruction were evaluated in order to study the effect of the microstructure in the in vitro behavior. Scanning electron microscopy, X-ray diffraction, Fourier transform infrared spectrometry, mechanical resistance of blocks, mercury porosimetry analysis, in vitro bioactivity, and cell viability and proliferation were performed to compare the characteristics of both allograft materials against a synthetic calcium phosphate block used as a negative control. The herein presented results revealed a very dense structure of the low-porosity bovine bone blocks, which conferred the materials’ high resistance. Moreover, relatively low gas, fluid intrusion, and cell adhesion were observed in both the tested materials. The structural characteristics and physicochemical properties of both ceramic blocks (sintered and not) were similar. Finally, the bioactivity, biodegradability, and also the viability and proliferation of the cells was directly related to the physicochemical properties of the scaffolds.

## 1. Introduction

In severely atrophic jaws, reconstructive bone surgery needs block grafting, especially in cases where the resorption of the edentulous maxilla can create a reverse maxillomandibular relation or an increased vertical distance between the jaws [1]. In these situations, the main advantages presented by the technique using blocks are the possibility of confirming the grafted material to the receptor bed and the fact that it can be fixed by screws (generating greater stability to the graft).

Given the morbidity associated with using autografts, there is growing interest in developing animal or synthetic bone graft substitutes [1,2,3]. Although other kinds of a homologous graft (cadaver bone) have been proposed, concerns about the risk of contamination are substantial [4,5]. Efforts are being invested to develop alternative synthetic materials to recover or replace damaged tissues [6,7]. In this context, xenogenous and synthetic allogenic bone substitutes have been proposed as an alternative to autogenous grafts [8]. 

A good bone substitute should exhibit a similar morphology to a lost bone by displaying an internal architecture that allows the perfusion, adhesion, growth, and maturation of cells. It should also be highly hydratable to maintain an isosmotic environment that allows suitable mobility and growth of angiogenic factors, as well as low immunogenicity and being able to be fixed surgically with screws or wires. Besides that, the bone substitute should also be absorbed during a time that is compatible with bone formation [9,10,11]. Features that allow good cell intrusion and infusion (porosity and interconnectivity), as well as the chemical composition and mechanical properties resembling the lost bone characteristics, are desirable. Thus, scaffolds can be defined with a provisional matrix for bone growth by providing a suitable environment. The anatomy and macrostructure of the defect (receptor site) strongly influence biomaterial stimulation for new bone formation [1,2,7,12,13]. 

Hydroxyapatite ceramics manufactured from natural materials, such as coral or bone, shows the advantage that they inherit some properties of the raw materials such as the pore structure [14,15,16]. The xenogeneic cancellous bone of bovine origin with a structural and chemical composition comparable to human bone constitutes an alternative to meet many patients’ demands for grafts [2,7,10]. The manufacturing process of lyophilized bovine bone grafts should maintain the morphological structure and chemical nature of bone to produce a final product with excellent biomechanical characteristics. In this context, the bovine bone material should preserve the bone chemical structure (mineral–collagenous) and physical characteristics (porous trabecular) to provide successful bone deposition that promotes cell proliferation with osteogenic potential and new bone formation [17,18]. Bone substitutes materials should possess properties that allow the migration, differentiation, and proliferation of the cells involved in the repair process by producing the extracellular matrix required to regenerate lost or damaged tissue. Therefore, the biological activities of these synthetic materials are directly related to their physicochemical properties, such as ions composition, crystallinity, particle size, surface characteristics, and porosity architecture [2,19,20]. 

However, the process of treating materials for bone grafts, and their ideal composition, have been discussed and are subject to discrepancies. For example, the sintering process can either be used or not when preparing bone grafting substitutes to promote physicochemical effects in these materials [2,16,21]. Calcium phosphates (bioceramics) have been extensively researched and used as bone substitutes for their unique bioactive property and biocompatibility [7,22,23,24], which was why it was selected as the negative control. The degradation of materials derived from calcium phosphate takes place in vivo via dissolution and osteoclastic reabsorption process [25,26,27].

Some authors studied five commercially available bone graft blocks, DIZG (Advancing the world of tissue transplantation) Human-Spongiosa, Tutobone, Puros Allograft Spongiosa, OsteoBiol Sp, and Bio-Oss were histologically studied. Three out of the five bone blocks contained organic/cellular remnants, as revealed by histology stated that their blocks were free of such remnants. These remnants could result in an unexpected and undesired immune response, inducing a foreign body reaction, and thus compromise the safety, biocompatibility and function of these three bone substitute materials [28]. Tutobone has an excellent biocompatibility, good osteoconductive characteristics, and can be used for horizontal and vertical bone augmentation [29,30,31,32].

We compared these materials with a synthetic tricalcium-phosphate (TCP), evaluated as a negative control of the analysis process, by analyzing the porosity, chemical, and mineralogical composition and sample microstructure. Hence, the objective of the present study was to evaluate the effect of the microstructure (i.e., porosity, grain size, and phase composition), on the in vitro behavior of the two commercially available mineralized bovine bone blocks prepared by two different processes: sintered and not sintered, in a simulated body fluid, and the differential cell proliferation of a mouse preosteoblastic cell line (MC3T3 cells) growing on these surfaces.

## 2. Materials and Methods

### 2.1. Materials

The analyzed implantable materials are two commercial bone blocks graft substitutes of bovine origin: Orthogen Bone^®^ (Baumer SA, Mogi-Mirim, Brazil) called bovine bone 1, treated by a physicochemical process, e.g., the material was subjected to high-temperature deproteinization for sintering (950 °C), treated with organic solvent, and sterilized [27]. This processing removes the organic elements contained in intratrabecular spaces; and Lumina Bone^®^ (Critéria Indústria e Comércio de Produtos Medicinais e Odontologicos Ltda., São Carlos, Brazil), called bovine bone 2, treated by chemical processing to remove the vascular elements and adipose tissue contained in intratrabecular spaces, and to maintain the content of collagenous proteins (20–25%), and the mineral part composed of calcium and phosphorus. This product did not undergo the sintering step. All the samples were characterized with no previous treatment.

In addition, laboratory synthetic TCP was examined to compare it with those of the commercial samples, taken as a kind of negative control of the evaluation process proposed to present the completely different structures and origins of the tested commercial materials.

The laboratory synthesis of TCP was performed by a solid-state reaction from a stoichiometric mixture of anhydrous calcium hydrogen phosphate (CaHPO4, Panreac, Barcelona, Spain) and calcium carbonate (CaCO_3_, Fluka, Bucharest, Romania), which resulted in a granule of ~30 µm and a Ca/P ratio of 1.5. The CaHPO_4_ and CaCO_3_ mixture was prepared at high temperature (1000 °C) for 12 h before being slowly cooled. Finally, the structures obtained in the process were particulated and characterized by XRD (x-ray diffraction).

### 2.2. Materials Characterization.

The materials were first characterized by the mercury porosimetry analysis in the Poremaster-60 GT machine (Quantachrome Instruments, Boyton Beach, FL, USA) at a pressure between 6.600 KPa and 411,248.500 KPa to measure the porosity and pore size distribution, which resulted in the porosity having diameters ranging from 225 μm to 3.58 nm. Three samples of ~0.45 g with a 0.28 cc volume were analyzed by this technique. Other particles of the same group were used to measure if the porosity values presented differences above 5%.

To determine the exact density of granules, excluding the existing spaces (mass/volume) in each material in the solid state, helium gas picnometry was used (Multi-pycnometer, Quantachrome corporation, Bayton Beach, FL, USA). A minimum sequence of five series was tested for each material, and a minimum of four samples was tested per group. The mean values were compared with a significance level of 5%.

The physicochemical characteristics of the studied materials were evaluated by scanning electron microscopy (SEM, Hitachi S-3500N, Tokyo, Japan). Samples were quantitatively evaluated by the Electronic Dispersive X-ray Spectroscopy (EDX) model INCA 300 EDX Analytical System with detectors for the EDS analysis in SEM (Oxford Instruments, Abingdon, Oxfordshire, UK), which generated separate factors for an atomic number, absorption, and fluorescence. The microanalysis data were obtained from the mean of 10 independent determinations. Samples of all materials were triple-coated and metalized (Polaron K550X Sputter Coater, Darmstadt, Germany) because they were non-conductive materials.

The crystallinity and composition phase characteristics of each studied material were determined by X-ray diffraction (XRD-Bruker-AXS D8Advance, Bruker, Karlsruhe, Germany).

To determine the size of the crystals present in each material, the Scherrer formula was used by taking the XRD standard as a reference. The lattice strains were disregarded because they were relatively small for the magnification corrections of the instruments.

$$D_{\mathrm{hkl}\;}=\frac{k\lambda }{B_{1/2}\cos\theta_{\mathrm{hkl}}}$$

Description factors: k is 0.89; λ is the wavelength of Cu Kα1 (λ = 1.54056 Å); B_1/2_ is the full width at a half maximum (rad) for (hkl) reflection; θ_hkl_ is the diffraction angle (°). The line broadening of the (211) reflection that corresponded to the maximum intensity peak was used to evaluate crystal size.

The following equation was applied for the crystallinity degree of the materials. The crystallinity degree of the as-dried samples (XC) equaled the fraction of the crystalline phase present in the examined volume, which was evaluated by the relation [33]:

XC ≈ 1 − (V x/y/Iz)

where Iz represents the reflection of hydroxyapatite in 300 and the tricalcium phosphate in 101 and V x/y is the reflection of empty spaces value between 112 and 300, which completely disappeared in the non-crystalline samples for HA (hydroxyapatite) in 128, and in 101 for TCP.

Another check was made of crystal size, applied using the following method:

Bx (Xc)1/3 = K

where K represents the constant found (0.24) used for the majority of the different calcium phosphate powders, and Bx is the full-width at a half maximum (FWHM) (in degrees) of the reflection (002) for HA and (101) for TCP.

The cell parameters of the HA and TCP phases were determined using the FPM fit algorithm form Bruker EVA 2 software (Bruker Corporation, Billerica, MA, USA) for each ceramic. The reference for HA was JCPDS no. 09-0432 (a = b = 9.418 Å, c = 6.884 Å, space group P63/m, theoretical density 3.156 g/cm^3^, Z = 1). The reference for TCP was JCPDS no. 55-0898 (a = b = 10.426 Å, c = 37.376 Å, space group R-3c, theoretical density 3.466 g/cm^3^, Z = 1).

To determine the chemical composition and major functional groups, Fourier transforms infrared spectroscopy was used (FTIR-ThermoNicolet IR200, Waltham, MA, USA). The FTIR spectra were recorded between 400 and 4000 cm^−1^ at 2 cm^−1^ resolution. The particles of each sample were prepared by adding the KBr matrix at a level of 1 wt%. The background data were collected for the KBr matrix and subtracted from each spectrum, which was recorded at ambient temperature. The collagen for laboratory use (Sigma–Aldrich, Steinheim, Germany) was used to compare its spectra with those of the graft materials.

**Figure 1:**
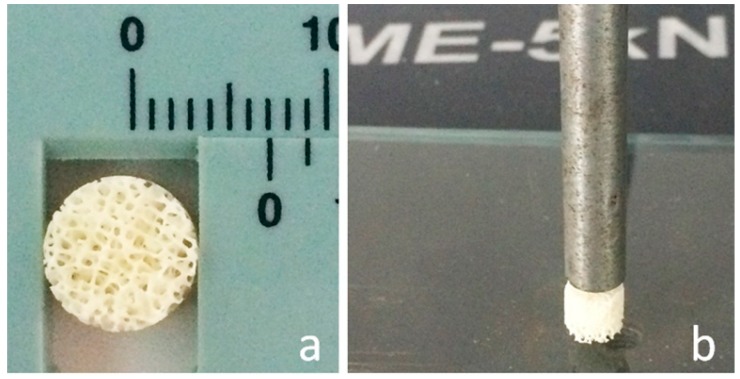
The mechanical test used in this work: (**a**) the block dimension after the cut; (**b**) the Diametric Compression of Block Test (DCBT).

The strength analysis was performed only on the blocks samples because the TCP samples had a very different size and were too small to be compared to the blocks in this type of test. To guide this test, the Brazilian test, the diametric compression of discs test (DCDT) [34,35], and the standard ASTM F1538-03(2017) [36] were used. Testing was conducted on cut cylindrical blocks (*n* = 10 per group), 7 mm in diameter × 10 mm long (Figure 1). Samples were positioned between the load application axis and a base plate by emitting axial forces in a universal test machine (model AME-5 kN, Técnica Industrial Oswaldo Filizola Ltda., São Paulo, Brazil) at a speed of 1 mm/min. Ten samples per group were analyzed and mean strength was performed. To statistically compare the data of this analysis, the *t*-test was used (*p* < 0.05). The power analysis of samples was 0.95 and the effect size was 2.66.

### 2.3. In Vitro Assays

The in vitro bioactivity of materials disc was assessed by soaking samples in SBF solution, which was prepared according to the procedure described by Kokubo et al. [37]. Discs, which measured 6 mm in diameter and 2 mm in thickness, were cut and washed with pure acetone before being hanged with a nylon thread in polystyrene tubes that contained 100 mL of SBF (pH 7.25). The solution was refreshed with 25% of fresh SBF every 24 h. The tubes with SBF and samples were incubated at 37 ± 0.5 °C in a shaking water bath for predetermined intervals. After different soaking periods that lasted from 3 days to 14 days, discs were removed from the SBF solution, gently washed 3 times with double-distilled water and dried for 24 h at room temperature. Any morphological variations in the disc surface were analyzed by SEM-EDS.

To evaluate the dissolution of the scaffolds, materials were soaked in 100 mL Tris-HCl solution (pH 7.40) at 37 °C for 14 days, and the solution was refreshed with 25% of fresh Tris-HCl every 24 h. Tris-HCl was selected because it does not contain inorganic ions (e.g., Ca, P, and Si). The weight of the discs before soaking was ~0.245 g. After the set soaking time, ceramics were dried at 120 °C for 1 day, and the final weight of each sample was accurately recorded. Weight loss was expressed as the percentage of initial weight. Ten samples of each material were used for this test.

For the cell viability and proliferation analyses, ten blocks of each test group and ten 0.10 g TCP granules were deposited into 96-well plates (one block/granules per well). Moreover, ten empty wells were reserved for control assays. For proliferation assays, the Pre-osteoblastic MC3T3-E1 Subclone 4 cell line (American Type Culture Collection, Manassas, VA, USA) was used. Cells were cultured in α-modified minimum essential medium (α-MEM, Nutricell, São Paulo, Brazil) supplemented with 10% fetal bovine serum (FBS, Invitrogen, Carlsbad, CA, USA) and 1% antibiotic/antifungal (PSA, Cultilab, São Paulo, Brazil) at 37 °C in 5% CO_2_ atmosphere. Confluent cells were trypsinized, washed with α-MEM, and counted. Afterward, MC3T3 cells were incubated at a density of 1 × 10^4^ cells/mL on each sample surface for 3 days at 37 °C in 5% CO_2_ atmosphere, and culture medium was changed daily. Cell proliferation was assessed by the MTT colorimetric assay after 3 days of culture as previously described [38]. Briefly, adhered cells were washed with phosphate-buffered saline (PBS) and 20 microliters of MTT (3-(4,5- dimethylthiazol-2yl)-2,5 diphenyl tetrazolium bromide, Sigma–Aldrich, St. Louis, MO, USA) were added to each well in a 5 mg/mL concentration. After 3 h of incubation at 37 °C, at 5% CO_2_ the supernatant was removed and 150 ml of dimethyl sulfoxide (DMSO, Sigma–Aldrich, St. Louis, MO, USA) were added to each well to release the formazan produced inside the cells by mitochondrial dehydrogenases. The absorbance of the resulting colored solution in each well, which represented the number of viable cells, was measured in a spectrophotometer (EL800, Bio-Tek, Winooski, VT, USA) at a wavelength of 570 nm.

## 3. Results

### 3.1. Results of the SEM and EDS Analyses

**Figure 2:**
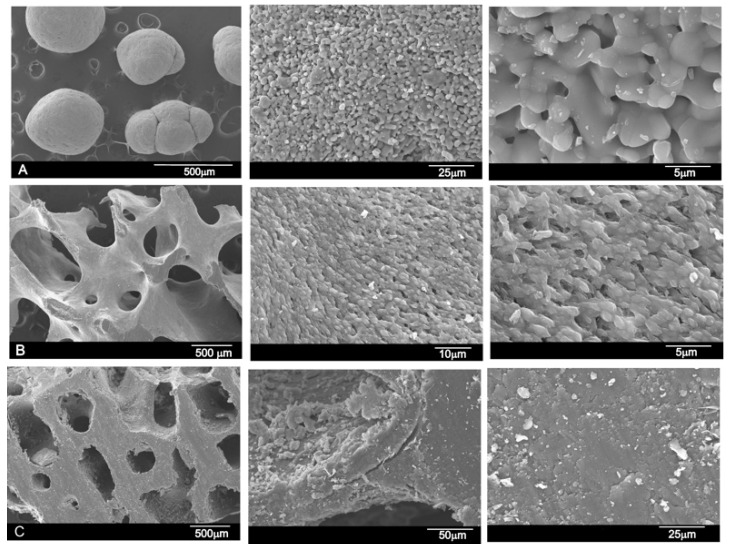
SEM micrographs of the different grafting materials: (**A**) synthetic TCP used as the control material; (**B**) bovine bone 1 (sintered material); and (**C**) bovine bone 2 (material not sintered).

The microstructural characterization of the grafting materials assessed by electron microscopy is shown in Figure 2. The synthesized TCP was prepared in the laboratory and was formed almost by spherical particles (500–1000 µm in size). The TCP surface was characterized by a rough morphology. With larger increments, the material showed an open microstructure with the stretching of pores (Figure 2A). The bovine bone 1 scaffolds were highly porous with a large pore size of ~500–700 µm (Figure 2B). The bovine bone 2 scaffold also presents porosity, although pore size was approximately half that of bovine bone 1 (~200–400 µm) (Figure 2C). With larger increments, the material presents a smooth surface with no porosity at the SEM magnification level. In the higher magnification images, the tested bovine bones do not show as much porosity as the TCP structure, which agrees with the results in Table 1, where the total porosity is around 20%.

The chemical surface composition of the three materials (TCP, bovine bone 1, and bovine bone 2) was analyzed by EDS in different areas of the same sample. In the TCP samples, CaO was 54.22 ± 7% and 45.64 ± 7% of P_2_O_5_, with Mg as the major impurity, which is commonly found in commercial calcium phosphate compositions. Bovine bone 1 presented 25.66 ± 5% of CaO and 27.51 ± 5% of P_2_O_5_, whereas bovine bone 2 presented 26.37 ± 4% of CaO and 26.93 ± 5% of P_2_O_5_. In both tested samples, the major impurity was Al, with 45.32% for bovine bone 1 and 44.76% for bovine bone 2.

### 3.2. Results of the Fourier Transform Infrared Spectroscopy (FTIR) Analysis

**Figure 3:**
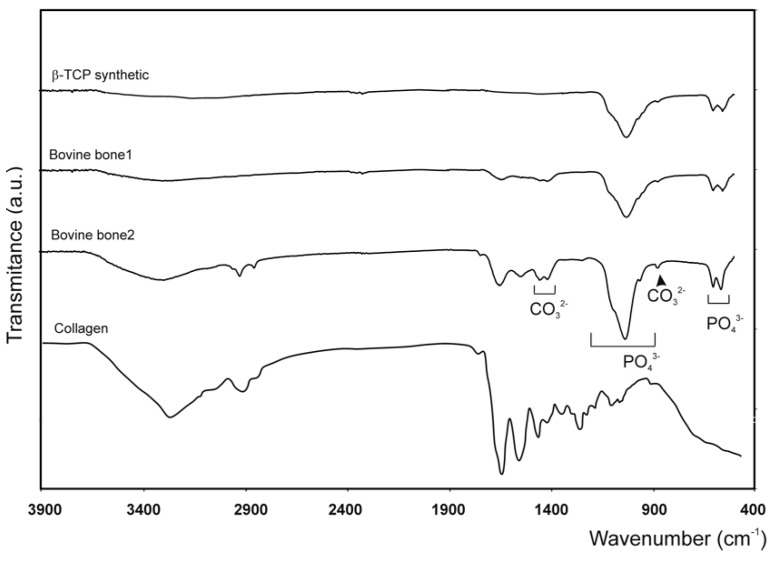
The FTIR spectra of the three tested synthetic materials (TCP, bovine bone 1, and bovine bone 2), plus a collagen matrix.

Fourier transform infrared spectroscopy is an ideal technique to analyze the chemical structural properties of natural materials since the frequencies of several vibrational modes of organic and inorganic molecules are active in infrared. This analysis confirmed the similarity in the composition of both the tested bovine bone models, with no differences due to them being sintered or not. As expected, the bovine bone samples gave lower values due to the collagen content in the material. Predictably, the commercial biomaterials showed the typical bands caused by hydroxyapatite, which was the major portion of the components in bovine bone: 1125–1040 cm^−1^ (ν3); 963 cm^−1^ (ν1), and between 550 and 610 cm^−1^ (ν4). More intense phosphate stretching bands can be observed at around 1043 cm^−1^ and 1092 cm^−1^. Both the commercial bovine bones also present a double band at 1420–1460 cm^−1^ (ν3) and a low-intensity band at 882 cm^−1^ (ν2), which represent the stretching vibrations of CO_3_^2−^ by replacing the PO in the apatite lattice. The FTIR spectra of both the bovine bone blocks and the TCP material (control), as well as the spectrum of collagen used as a comparative control, are shown in Figure 3.

### 3.3. Results of the X-ray Diffraction (XRD) Analysis

**Figure 4:**
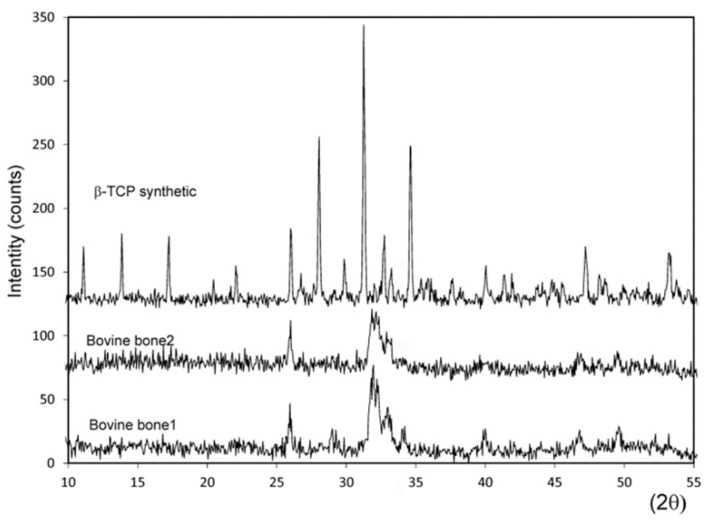
X-ray diffraction (XRD) of the synthetic TCP, bovine bone 1, and bovine bone 2.

X-ray diffraction was used to characterize the structural properties, crystalline phases, crystal size, and crystallinity of the three materials. Based on the X-ray diffraction (XRD) patterns, the crystal size of the bovine bone 2 ceramic resulted in 181 Å, with an estimated crystallinity degree value of about 30% and calculated cell parameters of a = b = 9.410 Å, c = 6.888 Å. Bovine bone 1 presented higher and better-resolved peaks, which indicate a material with 41% crystallinity, calculated cell parameters of a = b = 9.399 Å, c = 6.874 Å, and a crystal size of 217 Å. The XRD of the synthesized TCP powder showed sharp and well-resolved peaks compared to the natural ceramics, which corresponded to a highly polycrystalline material with 59% crystallinity and a crystal size of 1091 Å. The calcium phosphate used as the control material (TCP) had calculated cell parameters of a = b = 10.431 Å, c = 37.345 Å. No other phases were found. Figure 4 shows the X-ray diffraction patterns of both the bovine bones and the TCP material.

### 3.4. Results of the Mercury Intrusion Analysis

**Figure 5:**
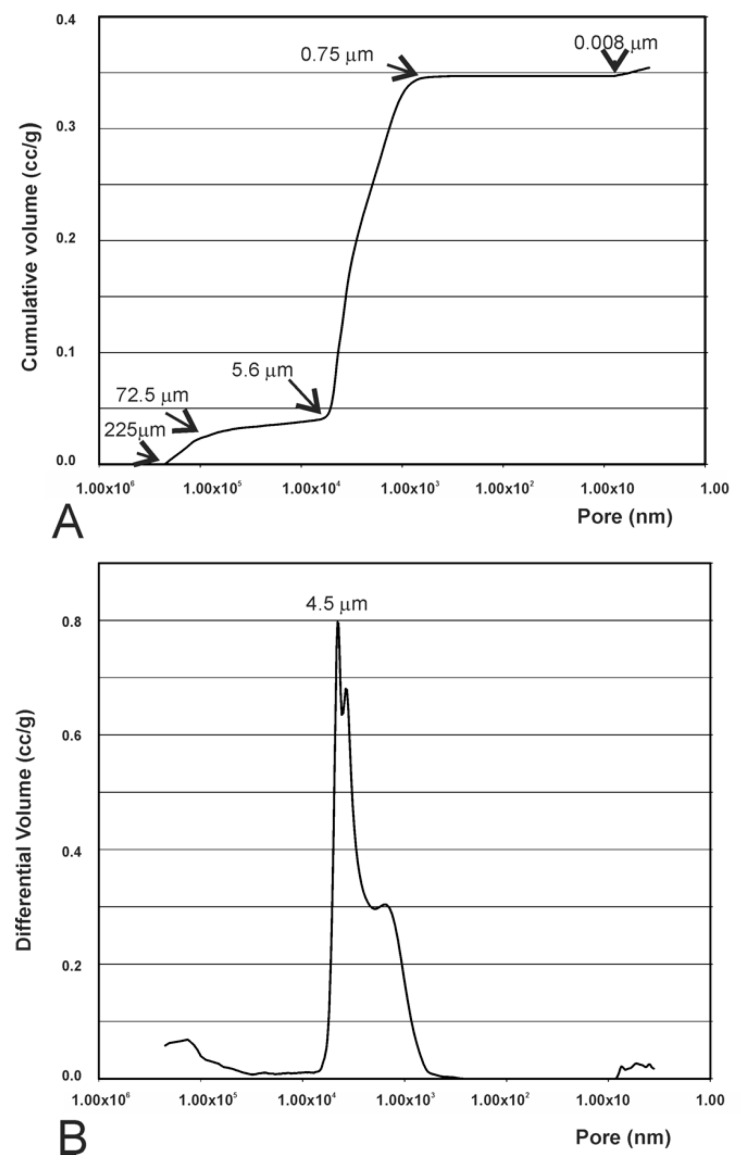
(**A**) Mercury intrusion curves of the synthetic TCP measured by mercury porosimetry: cumulative intruded volume vs. pore diameter and (**B**) differential-intruded volume vs. pore diameter.

The mercury intrusion porosimetry measurement showed that pores were interconnected, and that pore sizes ranged from several microns to hundreds of microns. The values measured for the synthetic TCP particles (Figure 5) demonstrated that, by increasing pressure, Hg intruded even where porosities were very small. The cumulative curve (Figure 5A) showed slight intrusion in the porosities between 225 μm (highest detected limit) and 72.5 μm, accompanied by a level between 72.5 and 5.6 μm with no intrusion at all. In the latter part of the curve, minor Hg intrusion took place with pores smaller than 0.008 μm. There was a correspondence between the initial increase in the curve and the filling of the porosities between particles, and the final increase stage was linked to the porosity of particles individually. In Figure 5B we can more easily observe the variation in intraparticle porosities, where a more intense peak of approximately 4.5 μm is clearly noted, which represents the majority of intraparticle porosities. The dimensions of these porosities, linked to the way in which these particles were involved, would surely depend on the dimensions and distribution of particles.

**Figure 6:**
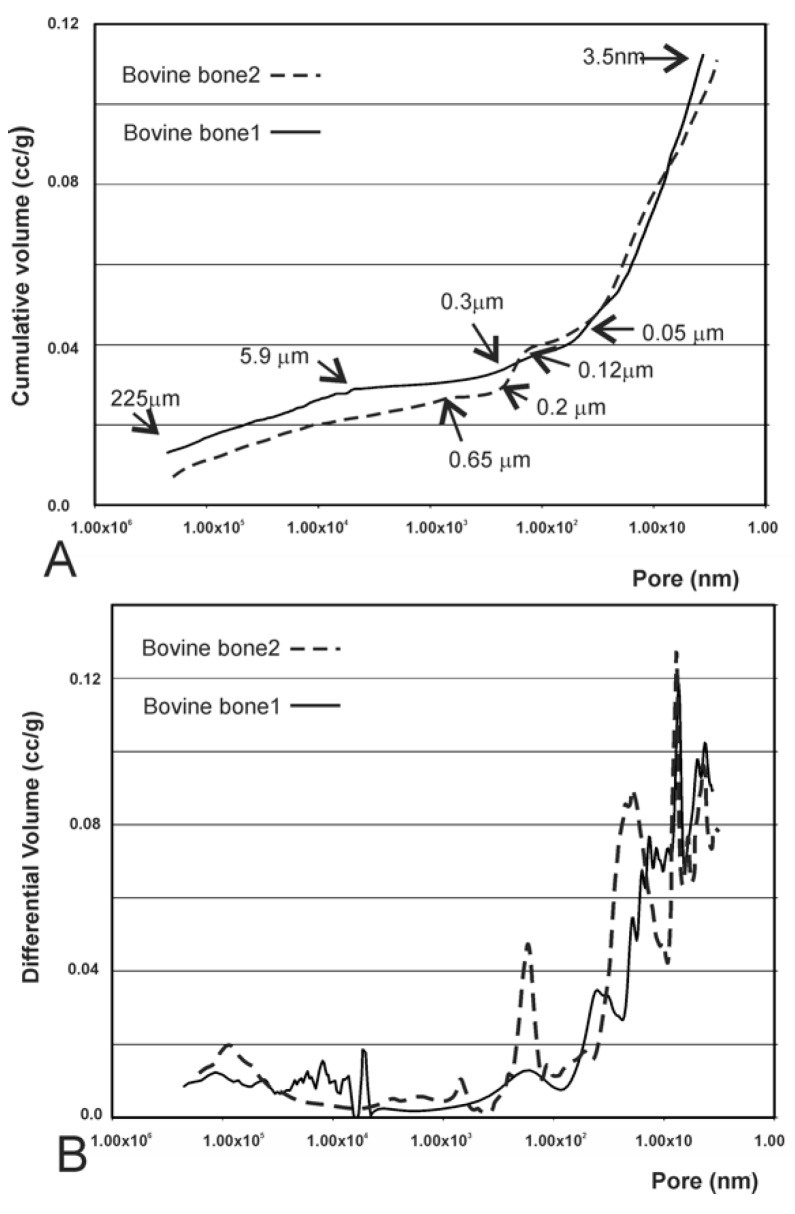
(**A**) Mercury intrusion curves of bovine bone 1 and bovine bone 2 measured by mercury porosimetry: cumulative intruded volume vs. pore diameter and (**B**) differential-intruded volume vs. pore diameter.

Both the bovine bone scaffolds presented high porosity (see Figure 2B,C), which exceeded the upper limit of detection of mercury porosimetry. The mercury intrusion curves revealed the presence of interparticle and intraparticle pores (Figure 6A). Bovine bone 1 showed a minor intrusion in pores from 225 µm to 5.90 µm, followed by a plateau to 0.31 µm in which no intrusion was detected. This was, in turn, followed by a significant intrusion of Hg up to 3.5 nm (under the detected limit). Bovine bone 2 showed three gas intrusions, 225 µm–0.65 µm, 0.22–0.12 µm, and 0.05–0.0035 µm, where the first corresponded to interparticle and the last two corresponded to intraparticle. The behavior noted within the intraparticle pores range was similar in both materials (Figure 6B), with different gas intrusion peaks unlike the synthetic TCP material (Figure 5B).

### 3.5. Results of the Helium Gas Pycnometry Analysis

The real density of particles (sample mass/volume of the solid; excluding empty spaces) was determined by He gas pycnometry. This measuring method excludes sample interstices and most pores since the small volume of gas molecules (He) enables their intrusion in almost all empty spaces. By helium gas pycnometry (Table 2), major density values were observed for the synthesized TCP compared to the bovine bone blocks. This value came close to the theoretical density of the tricalcium phosphate crystal (Ca_3_(PO_4_)_2_), reported to be 3.14 g/cm^3^.

### 3.6. Results of the Compressive Strength Analysis

**Figure 7:**
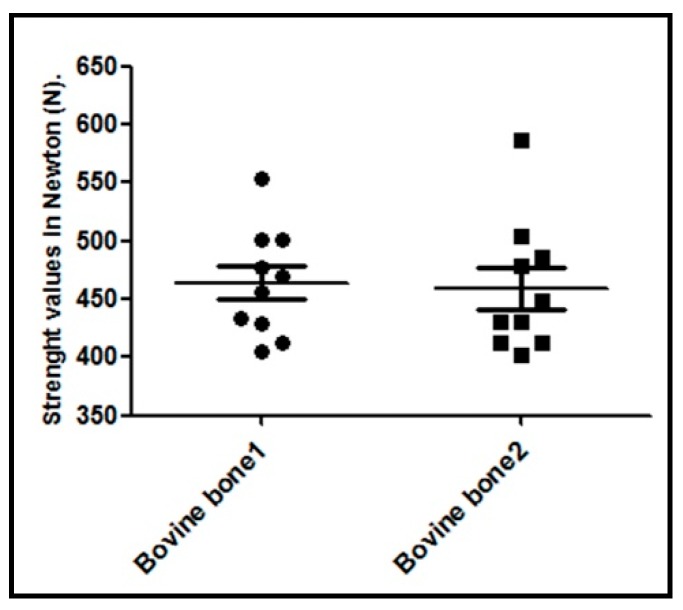
Graph of the compressive force values, standard deviation, and median of the measures for each sample block of both the proposed groups.

The property that is most often used to characterize the mechanical behavior of bone substitutes is their compressive strength. Failure occurs by the rupture and fragmentation of blocks into small pieces. The maximum load value supported by each sample was recorded and is shown in the graph of Figure 7. The collected values did not show any significant statistical intergroup differences (*p* = 0.8797).

### 3.7. Results of Bioactivity and Biodegradability Assays.

**Figure 8:**
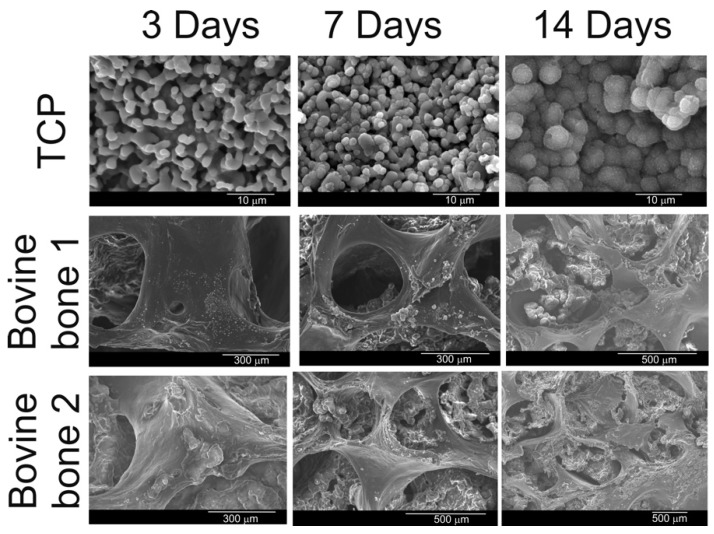
SEM micrographs of the scaffolds soaked in SBF for 3, 7, and 14 days.

Figure 2 and Figure 8 respectively show what the surface of the scaffold looked like before and after SBF testing. After the experiment, the surface of the TCP was covered by a layer of globular particles of about 3–5 µm in diameter (day 14), and the layer covered the entire surface of the specimen after 7 days. The EDS analysis of the layer revealed Ca/P ratios to be ~1.86, which were higher than that in the HA stoichiometric. The bioactivity of both bovine bones was not as obvious as the TCP scaffold. Bovine bone 1 present at third-day small globular precipitate scattered on the surface of the scaffold. As time passed, the globular deposits grew somewhat in size (~2–3 µm) forming small clusters scattered on the surface of the material that never came to form a compact layer (day 14). The EDS analysis of the precipitate give a Ca/P ratio of 1.94. This fact suggested that a Ca-deficient hydroxyapatite (CDHA) formed on the scaffolds’ surface. For the same period of time, no precipitate was observed for the bovine bone 2 material. It is worth noting that both bovine bones have in common a crumbling of the 3D structure, being more evident in bovine bone 2 at day 14.

On the other hand, both bovine materials had a high dissolution rate: bovine bone 1 had at the end of the experiment a weight loss of 1.2%, while bovine bone 2 had a weight loss of 3.7 %. This loss of weight can be correlated with the dissolution or disintegration of the 3D structure presented by both bovine bones at the end of the experiment (Figure 8, day 14).

### 3.8. Results of the Viability and Proliferation of Cells

**Figure 9:**
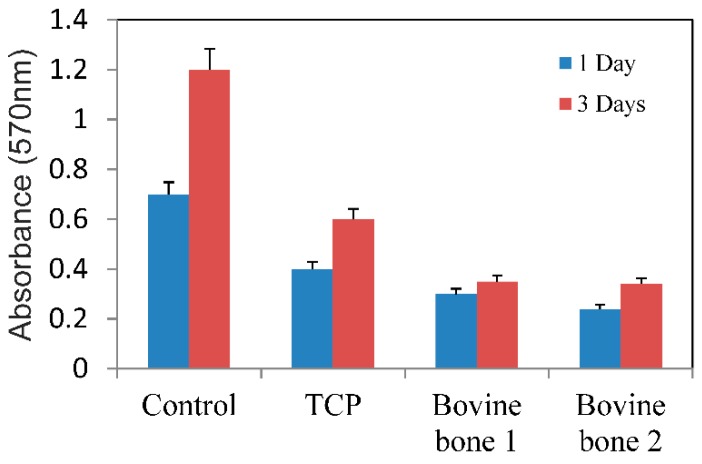
Viability assay of the cells growing in both test materials (bovine bone 1 and 2), TCP, and control plate assessed by the MTT bioassay. The mean values of absorbance at 570 nm are represented. The mean values are expressed as a percentage ± standard deviation.

After 3 incubation days, the MTT assay showed low cell proliferation (Figure 9) for the MC3T3-E1 cells in both the bovine blocks groups compared to the control groups. After 3 days, no difference was noted between the two test groups (*p* = 0.6983).

## 4. Discussion

Tissue engineering strategies include the introduction of a natural or synthetic biomaterial developed in an attempt to replace parts of tissues lost for different reasons, mainly in accidents or due to degenerative diseases. When used as a delivery vehicle for cells, biomaterials must provide a suitable microenvironment for cell survival, tissue regeneration, and host tissue integration. This potential biological capacity is related directly to the physicochemical properties presented by the material. The particulate materials for bone grafts, in many cases, are favored because they fill sufficiently irregular intraosseous defects [1]. However, these particulate materials may escape from the grafted site, and thus additional materials and methods such as a barrier membrane are needed to place the grafted materials in the proper position and avoid a collapse of the grafted site. The use of a barrier membrane involves additional expenses and prolonged surgical time, exposing patients to greater discomfort. Thus, bone substitutes with a good space maintenance capacity (in blocks form) without any additional materials are more appropriate for better clinical outcomes [39]. Although these materials are marketed in some parts of the world, few independent scientific studies are found in the literature on bone grafts in blocks. In this sense, different laboratory tests were performed on two bovine bone blocks manufactured for bone regeneration in humans, where the basic difference between these tested materials lies in the sintering process. Our results revealed that a sintering temperature of 950 °C (bovine bone 1) did not modify phase stability, densification behavior, fluid intrusion, and porosity compared to the similar bovine bone block that had not been sintered (bovine bone 2). Other studies have shown that high-temperature sintered biomaterials can lead to non-absorbable products [40,41].

The chemical characteristics of the tested materials were associated directly with the XRD results, in which the commercial samples of bovine bones presented a pattern that corresponded to hydroxyapatite, with the moments of peak and relative intensities coinciding. The tested samples of the different materials showed varying degrees of crystallinity, as indicated by different peak widths. The diffraction analysis demonstrated wide peaks for bovine bone 2 (not sintered), with a low signal-to-noise ratio. Low crystallinity and crystallinity degree of about 30% were determined for this material, whereas bovine bone 1 (sintered at 950 °C) presented more marked and better-resolved peaks, which indicates a material with 41% crystallinity. The XRD of the synthesized TCP powder showed sharp well-resolved peaks compared to the natural ceramics, which corresponded to a highly polycrystalline material with 59% crystallinity. Crystallinity is highly dependent on sintering temperature because a high sintering temperature results in a more perfect crystal, thus the degradation rate lowers [42,43,44].

The commercial bone substitutes showed the typical bands caused by hydroxyapatite, which constituted the major portion of the components in the bovine bone: 1125–1040 cm^−1^ (ν3); 963 cm^−1^ (ν1) and between 550 and 610 cm^−1^ (ν4). More intense phosphate stretching bands appeared at around 1043 cm^−1^ and 1092 cm^−1^. Also stretching vibrations of CO_3_^2−^ when replacing PO in the apatite lattice [45]: double band at 1420–1460 cm^−1^ (ν3) and a low-intensity band at 882 cm^−1^ (ν2). Values above 1300 cm^−1^ represented the bands that are most often referred to as collagen vibrations, except for those that came about from CO_3_^2−^ at 1423 cm^−1^ and 1456 cm^−1^ [46], and the broad bands present at 3500 and 1657 cm^−1^, thus designated by the present OH structural groups; i.e., by the sequence of the flexing mode of the H–O–H groups and by the elongation mode between the O–H groups [47].

The spectra of TCP only showed bands related to PO42-groups. All the bands found in the TCP spectrum were associated with phosphate, and no carbonate or hydroxyl groups appeared. In this context, the symmetric and antisymmetric stretching modes of the phosphate group were highlighted by the following bands: the band from 963 cm^−1^ was attributed to symmetric stretching mode ν1 [48], while the band from 1092 cm^−1^ was attributed to asymmetric stretching mode ν3 [48]. The bending modes of the phosphate group were evidenced by the bands from 565 cm^−1^ and 605 cm^−1^, respectively. Both were attributed to mode ν4. Furthermore, the band from around 1043 cm^−1^ was associated with bending mode ν3.

Distinguishing the porosities between and within particles can sometimes be difficult. The purpose of this information is to determine and/or interpret the signals obtained on the distribution curves of porosity size to be able to specify the amplitude of measured porosity. In the present study, the limit between intra- and inter-porosity was established at about 5 µm. The mercury intrusion porosity analysis particularly showed porosity within particles and was less suitable for measuring large spaces such as interparticle spaces (225 µm).

Regards to the resistance of the blocks, the high strength values presented by the two tested materials (bovine bone 1 and bovine bone 2), as generally found for highly dense structures, resulted from the low porosity in these materials, which was corroborated by the results obtained in the present study. Other authors have reported that large porosities certainly reduce a material’s mechanical strength and may alter the cellular processes involved in tissue healing, e.g., in new bone formation in this case [49,50]. Then, a larger pore can affect the stability of the scaffold and its ability to provide physical support for the seeded cells [51,52]. Regarding mechanical proprieties for biomaterials, in 2011 Scarano et al. [53] demonstrated that the rigidity of the block was able to maintain adequate space for bone regeneration and did away with using mini-screws and mini-plates for stabilization purposes.

Although the structural arrangement of biomaterials may have control during the technological manufacturing process and subsequent physicochemical treatments [50,51], such as sintering, the properties, and morphology of these materials can be considerably modified, which is directly related to the treatments and/or products applied to the materials. Both pore morphology and size are affected by high temperatures when applied to sintering. Moreover, with a change in the specific surface area, density and porosity, the material metabolism properties (dissolution and/or resorption) during the subtraction process can be strongly affected. When high temperatures are used, physical properties such as density, particle size, compressive, strength, and torsional force can significantly alter [2,15,19,20]. However, the results obtained in our study showed that there was no significant difference in any of the parameters tested between bovine bone blocks sintered at 950 °C and not sintered.

The rate of the formation of HA-like layer is a measure of bioactivity and the HA-like formation ability is thought to be a critical factor in facilitating the chemical fixation of biomaterials to bone tissue, and ultimately the in vivo success of the bone grafting material. The mechanism of HA-like phase can be explained in terms of a chemical reaction taking place between the Ca–P materials and the solution.

When immersed Ca–P material in SBF solution, both dissolution, and HA-like precipitation occur on the surface on the materials. At an early stage, the dissolution of Ca–P generally proceeds faster than precipitation. When the materials get in contact with the SBF a partial dissolution occurs producing an ionic exchange of Ca^2+^ for 2H^+^ within the material network leading to the formation of crystallization nuclei for the Ca–P phase which can be formed from the high concentration of Ca and P present in the medium.

In the beginning, the TCP dissolves slightly, so at day 1 we can better see the edges of the TCP grains (Figure 8). Over time the TCP reacts with the Ca and OH ions present in the medium which becomes HA-like according to: 3[Ca_3_(PO_4_)_2_] + Ca^2+^ + 2OH^−^ → Ca_10_(PO_4_)_6_(OH)_2_, and leads the nucleation on the surface of the globular particles of Ca–P powders that by that time transforms into a new Ca–P rich layer. This precipitate was growing with exposure time (Figure 8, 14th day), developing a continued layer.

On the other hand, in bovine bone 2, the dissolution of the material was the dominant process throughout the experiment and no precipitation took place (3.7% of the weight was lost at the end of the assay). The bovine bone 1 with an intermediate behavior showed some precipitation, but the dissolution of the material was still the dominant process (1.2% of the weight was lost). This behavior can be correlated with the crystallinity of the materials.

The precipitation process was dominant in high crystalline a material and the dissolution process will be dominant in materials with poor crystallinity. The mechanism of bioactivity is a competitive process of dissolution–precipitation where it is possible to model the behavior of the materials changing the physicochemical characteristic of the graft materials through the manufacturing conditions, so we can design materials with specific needs.

The biological behavior of the materials can be studied by cells cultures over your surface. In this way, several researches have been carried out to investigate the interaction between cells and biomaterials [51,52]. In this sense, the surface morphology is also an important factor to affect the cell behaviors [54,55]. To date, cell seeding on 2D scaffold, surfaces have been shown to be easy to perform but the preparation of 3D cell-scaffold constructs for regeneration of organs is far more complex. For example, pores of adequate size allow cells to migrate or adhere to the surface of a material, but interconnecting pores are necessary to permit cell growth into the scaffold interior. A common problem encountered when using scaffolds in tissue engineering is the attachment and proliferation of the rapid cells on the outer edge of the scaffold which restrict cell penetration to the scaffold center, resulting in a necrotic core [56]. In our study, the penetration of cells into the blocks was not tested, however, because of the results obtained with respect to the penetration of fluids, which were quite low; it is very probable that the cellular proliferation in these materials will be quite low. The low cell viability presented by the samples from both test groups (bovine blocks) corroborated these previous statements because as shown, both blocks have low porosity and low fluid penetration. A strategy to improve the results of these materials would be to cell seeding in the center of the scaffold [57]; however, the feeding the inner surfaces of the scaffolds are limited by the pores that are too small. After clinical evaluation of five bone blocks containing organic/cellular matrix demonstrated an excellent biocompatibility, good osteoconductive characteristics, and can be used for horizontal and vertical bone augmentation by the degradation and replacement less accelerated in patients than the animal study indicated [28,29,30,31,32].

Within the limitations of this study and in view of the results obtained, we cannot say which of the two materials (sintered or not sintered) is better. Each material can be used in a specific clinical application in reconstructive surgery of bone pathology. Bovine bone 1 (sintered), which represents the moderate dissolution pattern, allows implementation in situations requiring partial replacement by autologous bone with a matrix in place over the longer time. Bovine bone 2 (not sintered) with a faster dissolution rate allows its use in situations requiring rapid replacement by autogenous bone. The control material with of slow resorption pattern is suitable in the situation where dimensional stability of the implant is required.

## 5. Conclusions

In conclusion, the data from this study reveal that variations in physical properties, like phases, crystallinity, and porosity of calcium phosphate-based materials of either a natural or synthetic origin, strongly depend on manufacturing procedures. The bovine bone materials are monophasic with high porosity and medium-high crystallinity, except for bovine bone 2, whose crystallinity is lower due to the presence of a bigger quantity of collagen mixed with the hydroxyapatite matrix. As expected, the best crystallinity corresponds to the synthetic material. Pore size is similar for the studied materials of a natural origin, with certain advantages for the synthetic TCP material in relation to external pores and micropores interconnection. The viability and proliferation of the cells growing in direct contact with the bovine bone blocks are relatively low in comparison to the control group.

## Figures and Tables

**Table 1:** Mercury-intruded volume, mode (most frequent diameter) of intraparticle pores, total porosity, and intraparticle porosity of the commercial samples.

Biomaterial	Intruded Volume (cc/g)	Total Porosity (%)	Intraparticle Porosity (%) ^a^	Interparticle Porosity (%) ^b^
Bovine bone 1	0.1127	20.21	15.013	5.1969
Bovine bone 2	0.1119	19.28	16.29	2.9902
Synthetic β-TCP	0.3544	53.24	37.45	15.79

**^a^** Corresponding to 3.5 nm < pores < 5 µm; **^b^** Corresponding to 5 µm < pores < 225 µm.

**Table 2:** Real density measured by helium pycnometry and apparent density measured by mercury porosimetry.

Biomaterial	Real Density (g/cm^3^)	Apparent Density (g/cm^3^)
Bovine bone 1	2.25	1.79
Bovine bone 2	2.13	1.72
Synthetic TCP	3.22	1.50
